# Diversity of Pelagic and Benthic Bacterial Assemblages in the Western Pacific Ocean

**DOI:** 10.3389/fmicb.2020.01730

**Published:** 2020-09-10

**Authors:** Mengmeng Wang, Yiyuan Ma, Chunhui Feng, Lei Cai, Wei Li

**Affiliations:** ^1^College of Marine Life Sciences, Ocean University of China, Qingdao, China; ^2^State Key Laboratory of Mycology, Institute of Microbiology, Chinese Academy of Sciences, Beijing, China; ^3^Beihai Ocean Engineering Survey Research Institute, State Oceanic Administration, Qingdao, China

**Keywords:** bacterial community, 16S rRNA, seawater, sediment, open sea

## Abstract

Despite numerous studies on marine prokaryotes, the vertical distribution patterns of bacterial community, either on the taxonomic composition or the functional structure, remains relatively unexplored. Using HiSeq-derived 16S rRNA data, the depth-related distribution patterns of taxonomic diversity and functional structure predicted from diversity data in the water column and sediments of the Western Pacific Ocean were explored. The OTU richness declined along the water column after peaking between 100 to 200 m deep. Relative abundance of Cyanobacteria and SAR11 decreased significantly with depth, while Actinobacteria and Gammaproteobacteria increased. This clearly mirrors the vertical distribution pattern of the predicted functional composition with the shift between phototrophic to chemoheterotrophic groups from the surface to the deeper layers. In terms of community composition and functional structure, the epipelagic zone differed from other deeper ones (i.e., meso-, bathy-, and abyssopelagic zones) where no obvious differences were detected. For the epipelagic zone, temperature, dissolved oxygen, and salinity were recognized as the crucial factors shaping both community composition and the functional structure of bacteria. Compared with water samples, benthic sediment samples harbored unexpectedly higher read abundance of Proteobacteria, presenting distinguishable taxonomic and functional compositions. This study provides novel knowledge on the vertical distribution of bacterial taxonomic and functional compositions in the western Pacific.

## Introduction

Bacterioplankton are considered to play a central role in the biological cycle of carbon through the microbial loop (Stocker, 2012; Fuhrman et al., 2015). The knowledge of the distribution pattern of bacterial diversity and the relationship with hydrographic/environmental parameters is important for the understanding of their ecological functions. Previous studies indicated that bacterial richness and communities on the surface of water columns in the ocean vary from those in deeper levels (Delong et al., 2006; Durbin and Teske, 2011; Tseng et al., 2015; Zorz et al., 2019), as well as from those in seafloor sediments (Bienhold et al., 2016; Walsh et al., 2016). For example, Walsh et al. (2016) documented that Cyanobacteria, Flavobacteria, and Alphaproteobacteria dominated the photic-zone [above the deep chlorophyll maximum (DCM)] in water samples from the Pacific, whereas the aphotic-zone samples (below the DCM) were mainly dominated by Alpha-, Gamma-, Deltaproteobacteria, and Deferribacteres. However, marine sediments were generally represented by Proteobacteria, most of which affiliated with Gamma-, Alpha-, and Deltaproteobacteria (Bienhold et al., 2016; Walsh et al., 2016).

Depth is well-associated with a dynamic physical and chemical gradient in water columns (Stocker, 2012; Lønborg et al., 2016), which potentially results in a depth-related pattern of microbial distribution (Fuhrman et al., 2015; Guerrero-Feijóo et al., 2017). For example, the distributions of some bacteria (e.g., *Prochlorococcus* and SAR11) and Archaea follow depth stratification, which are suggestive of depth-variable adaptation to light intensity and nutrient availability (Delong et al., 2006; Yilmaz et al., 2016). Increasing evidence suggests that the relationship between prokaryotic species richness and depth is multivariate. For bacteria, some studies described an increase in richness with depth (Pommier et al., 2010; Kembel et al., 2011; Sunagawa et al., 2015; Walsh et al., 2016) while others observed a decrease (Brown et al., 2009; Agogué et al., 2012; Bryant et al., 2016). The discrepancy may depend on the different environmental setting of the sampling sites or the technical distinctions in the sampling and sample processing (Smedile et al., 2014). Therefore, more information on this topic, from various marine habitats, would be a valuable piece of the puzzle.

On the other hand, functional composition variation of microbial plankton in the ocean is found to be depth-stratified (Louca et al., 2016). Based on whole-genome shotgun sequencing, vertical zonation of protein functions was revealed in the water column of the Hawaii Ocean Time-series station (Delong et al., 2006). Compared to the sea surface, the deep-sea community had greater metabolic versatility and genomic plasticity to cope with the sparse and sporadic energy resources available (Konstantinidis et al., 2009). Using a similar method, Tseng et al. (2015) observed depth-dependent metabolic potentials in the water column of the South China Sea. Recently, Louca et al. (2016) developed a method to disentangle functional from taxonomic community variation by classifying marine microorganisms into metabolic functional groups. With this method, they described a depth-related trend of functional diversity of prokaryotic plankton in the global ocean from the surface to the mesopelagic zone. Despite these advances, our knowledge about the dynamics of prokaryotic functional structure in relation to depth, especially the influence of hydrographic parameters, is still limited.

The western Pacific is an ideal area to investigate the relationship between hydrography and distribution patterns of plankton communities, as it is constrained by strong and complex western boundary current systems that include the Kuroshio Current, Kuroshio Counter Current, Subtropical Counter Current, North Pacific Equatorial Current and North Pacific Equatorial Counter Current (Nagai et al., 2015; Liang et al., 2016). For example, distribution patterns of the zooplankton generally exhibit close relationships to those of water masses (Fager and McGowan, 1963). Moreover, the western Pacific is a hydrographically complex oligotrophic area (Liang et al., 2016). In the open sea, investigations on prokaryotic communities mainly focused on the marginal seas of China (e.g., Liu et al., 2014; Zhang et al., 2014; Tseng et al., 2015; Wang et al., 2015). However, the knowledge of large-scale patterns of prokaryotic diversity and community structure in the open sea of the western Pacific are still rather limited. Recently, using flow cytometry, Liang et al. (2016) described the dynamics of picoplankton in the western Pacific, and found that it was determined by a complex and depth-dependent relationship among physical and biological factors.

In the present study, water column samples were collected from seven locations in the western Pacific from the surface to a depth of 5,415 m. Using 16S rRNA metabarcoding, we examined the depth-related distribution pattern of taxonomic and functional diversity in the water column and the influence of hydrographic properties in the epipelagic zone. Moreover, five surface sediment samples from the Philippine Basin were also used to make a comparison between the planktonic and benthic microbial diversity.

## Materials and Methods

### Study Area, Sample Collection, and Oceanographic Parameters

**FIGURE 1:**
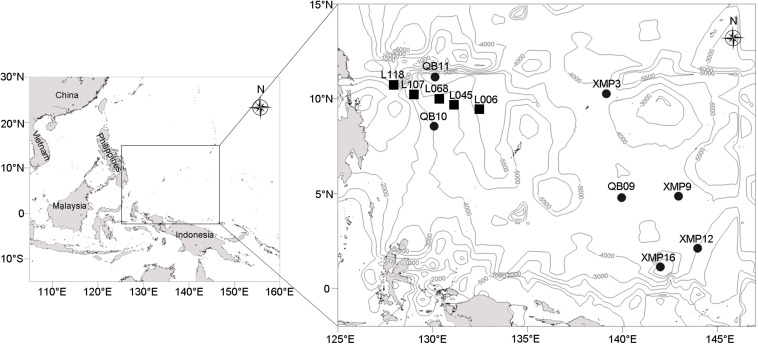
Localization of the seven water columns (black circles) and five sediment samples (black rectangles) in the western Pacific (depth contours are marked with gray lines).

Twelve stations located in the Western Pacific Ocean (Figure 1) were visited using the *Kexuehao* and *Xiangyanghong03* research vessels in December 2016. Water samples were collected from the surface to the near bottom of seven stations at 11–15 different depths according to water depth (Supplementary Table S1) using 25 L Niskin bottles mounted on a Seabird conductivity temperature depth probe (CTD) rosette. The 92 samples collected span four pelagic zones, i.e., epi- (0–200 m), meso- (201–1,000 m), bathy- (1,001–4,000 m), and abyssopelagic waters (4,001–6,000 m). Seawater samples (3 L) were filtered through 0.22-μm pore-size Sterivex membranes (Millipore, Billerica, MA, United States). Filters were then sealed and frozen at −80°C. Sediment was collected from the Philippine Basin (5,402–5,907 m water depth) using a 0.05 m^3^ stainless steel grab sampler (Wildco^®^, Florida, United States) at five stations. Surface sediments (2–5 cm depth) were transferred into 3.5 cm-inner-diameter sterilized glass tubes, which were sealed with sterile plastic film. Glass tubes were immediately stored at −80°C.

Ancillary data, including temperature, salinity, dissolved oxygen (DO), and dissolved nutrients (i.e., NO_3_^–^, PO_4_^3–^, and SiO_3_^2–^) of the epipelagic zone, were retrieved from the World Ocean Atlas 2013 version 2 (WOA13 V2) (Boyer et al., 2013), where only December data in the 2005–2012 period were considered. A one-degree grid was chosen for spatial resolution of DO and dissolved nutrients, while a quarter-degree grid was selected for temperature and salinity. Lagrangian interpolation (Reiniger and Ross, 1968) was applied to resolve vertical distribution parameters at 5 m intervals using 37 standard depth levels in WOA13.

### DNA Extraction, Library Construction, and High-Throughput Sequencing

Genomic DNA was extracted according to Collins et al. (2010) with optimizations. Filters were cut into pieces using sterile scalpels under a laminar flow and transferred into Matrix E lysing tubes (MP Biomedicals, Solon, OH, United States) that already had beads in them. After the addition of 0.2 g of cell breaking beads, 400 μL of STE buffer (100 mM NaCl, 10 mM Tris-HCl, 1 mM EDTA, and pH = 8.0), and 80 μL of 20% SDS, tubes were homogenized twice in a FastPrep instrument for 40 s at a speed setting of 6.0 m/s, and then incubated at 65°C for 20 min. After centrifugation (14,000 *g* for 15 min), genomic DNA was concentrated using phenol-chloroform-isoamyl ethanol (volume proportion = 25:24:1), desalted using 70% ethanol, and then re-suspended in 50 μL of sterile ddH_2_O. Surface sediments (0.5 g) were removed from the glass tubes using a sterile spatula and mixed thoroughly. Genomic DNA extraction was performed on the slurry using a FastDNA^®^ SPIN Kit for soil (MP Biomedicals, Solon, OH, United States) according to the manufacturer’s instructions.

The V3-V4 region of 16S rRNA gene was amplified using the forward primer 341F (ACTCCTACGGGRSGCAGCAG) and reverse primer 806R (GGACTACVVGGGTATCTAATC) (Muyzer et al., 1993; Caporaso et al., 2012) that was supplemented with one of the 5-base identifier barcodes reported in Supplementary Table S2. The primers 341F/806R were widely used to characterize bacterial community in marine environments (Herlemann et al., 2011; Wang et al., 2015; Mestre et al., 2017). The reaction mixture consisted of 10 μL HotStarTaqMaster Mix (Qiagen), 500 nM of each primer, 10 ng of DNA, and nuclease-free water to bring the total volume to 20 μL. DNA was denatured for 15 min at 95°C, followed by 25 cycles of denaturation at 95°C for 20 s, primer annealing at 55°C for 30 s, and extension at 72°C for 30 s, followed by a final extension step of 72°C for 7 min. Five replicates of PCR products were pooled, and their relative quantity was estimated by running 5 μL of amplicon DNA on a 2% agarose gel. Products were purified with a GeneJET^TM^ Gel Extraction Kit (Thermo Scientific). Amplicon libraries were generated using a NEB Next^®^ UltraTM DNA Library Prep Kit for Illumina (NEB, United States) following the manufacturer’s recommendations, and index codes were added. High-throughput sequencing was performed on an Illumina HiSeq 2500 sequencing platform (Realgene Corporation, Shanghai, China) (paired-end reads 2 × 250 bp).

### Processing of Illumina Sequence Data

Paired-end raw reads were merged using PANDAseq (maximum mismatch ratio = 0.1, minimum overlap = 10 bp) (Masella et al., 2012) and reads less than 200 bp were removed. The command *split_libraries_fastq.py* of QIIME pipeline (Caporaso et al., 2010) was used to perform quality filtering (the options: −*q* = 30, −*r* = 3, −*p* = 0.75) and splitting libraries. Reads were further quality filtered using MOTHUR 1.33.3 (Schloss et al., 2009) with the following options: no DNA ambiguities allowed and minimum sequence length ≥220 bases. The HiSeq fastq files and the identifier barcode files were deposited in the National Center for Biotechnology Information Sequence Read Archive (SRA) as BioProject Accession no. PRJNA547878.

### Sequence Analysis, Taxonomic Identification, and Functional Prediction

UPARSE method available in USEARCH (Edgar, 2013) was used to cluster clean reads into operational taxonomic units (OTUs) at a sequence similarity threshold of 97%. Taxonomic assignment of representative sequences was performed with an 80% cutoff by applying MOTHUR 1.33.3 *classify.seqs* command (Schloss et al., 2009) using SILVA references (132 version).

Based on the identification and abundance information of the OTUs, we predicted their metabolic and ecologically relevant functions using the FAPROTAX database (Louca et al., 2016). In brief, a taxon table represented by the biom format was produced by the command pick_closed_reference_otus.py of QIIME pipeline (Caporaso et al., 2010) against SILVA references (132 version) with a similarity cutoff of 99%. Then, the taxon table was converted into a putative functional table represented by read abundance using the script collapse_table.py (Louca et al., 2016).

### Statistical Analyses

Using the SILVA online tool, i.e., TestPrime (Klindworth et al., 2013), we found that the pair of primers 341F/806R exhibited a high match percentage (83.1%) with Bacteria reference sequences in the SILVA database (132 version), whereas the pair exhibited a very low match percentage (0.3%) with Archaea. As expected, very low representation of Archaea (the fewest sequence per sample is one) was detected in our dataset. Subsequently, Archaea was removed from our dataset and only bacteria were treated in this study. To ensure inter-sample comparability, bacterial sequences were randomly resampled to reduce the number of reads in each sample to the lowest number of reads (25,345) in any individual sample. All further analyses were performed on the randomly resampled OTU table. For exploring community comparison among samples (i.e., beta-diversity), weighted UniFrac distance matrix was calculated using USEARCH (Edgar, 2013). Comparative analyses of α-diversity (OTU richness and Shannon index) between samples were conducted using the *vegan* package of R (version no. 3.2.3) (Oksanen et al., 2013). For analyses of ecological function structure, the relative abundance of a function group within each sample was estimated by comparing the number of reads assigned to each taxon for individual samples.

One-way ANOVA followed by Tukey’s “Honestly Significant Difference” method was used to explore variations in OTU richness and Shannon diversity among water zones. For the whole water column, multiple regression analyses were conducted to explore the correlations between bacterial diversity and depth. Pearson correlation coefficients and *p-*values were calculated to determine the associations between bacterial taxon diversity and environmental parameters. The principal coordinate analysis (PCoA) plots were used to visualize the ordinations of both the community and function structures based on weighted UniFrac distances and functional dissimilarities, respectively. The *adonis* function in the *vegan* package was run to test the influence of layer of the water column, depth, and station on community/function structures. In addition, a Mantel test or a partial Mantel test was chosen to check the correlations between dissimilarities and environmental variables {transformed by [log (*x* + 1)]}.

## Results

### Bacterial Richness Showed the Trend of First Rising and Then Falling

All samples showed high sequence coverage (25,345 minimum usable reads). In total, 2,458,465 reads (3,156 OTUs) could be assigned into 27 phyla, 56 classes, 134 orders, 238 families, and 378 genera (Supplementary Table S3).

**FIGURE 2:**
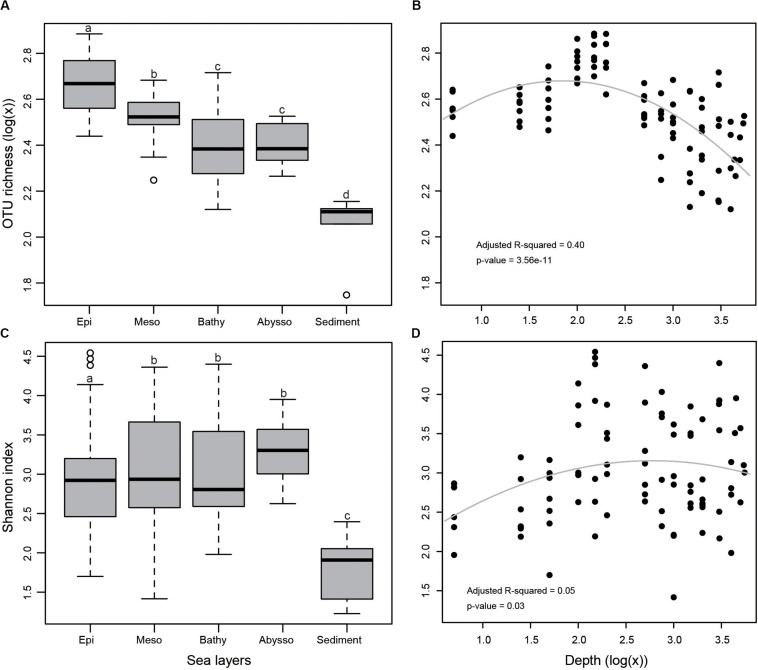
**(A)** Bacterial OTU richness in different zones as demonstrated by a boxplot with median and 95% confidence intervals, **(B)** multiple regression of OTU richness with depth, **(C)** Shannon index, and **(D)** multiple regression of Shannon index with depth. Bars without shared letters indicate significant differences at the level of adjusted *p*-value <0.05.

In the terms of both OTU richness (Figures 2A,B) and Shannon index (Figures 2C,D), sediment samples appeared to have lower diversity than that of water samples. Bacterial diversity was significantly higher in the epipelagic zone than in the deeper zones in terms of OTU richness (*p* < 0.05). From the surface water to the abyssopelagic zone, OTU richness appeared to have a decline trend after initial ascents along the water column (Figure 2B). Highest OTU richness always peaked between 100 and 200 m in water columns for all stations.

### Taxonomic Composition Switched Along Depth

In water samples, members of Proteobacteria dominated, accounting for 54.6% of total reads. They were mainly represented by Alpha- (26.0% of total reads) and Gammaproteobacteria (28.2%). Actinobacteria and Cyanobacteria were the second and third dominant phyla (18.5 and 12.5%) of the total reads, respectively. The remaining phyla of the water column consisted of Patescibacteria (7.1%), Firmicutes (2.9%), and Bacteroidetes (1.5%). In sediment samples, the bacterial community was overwhelmed by Proteobacteria (98.5% of the total reads).

**FIGURE 3:**
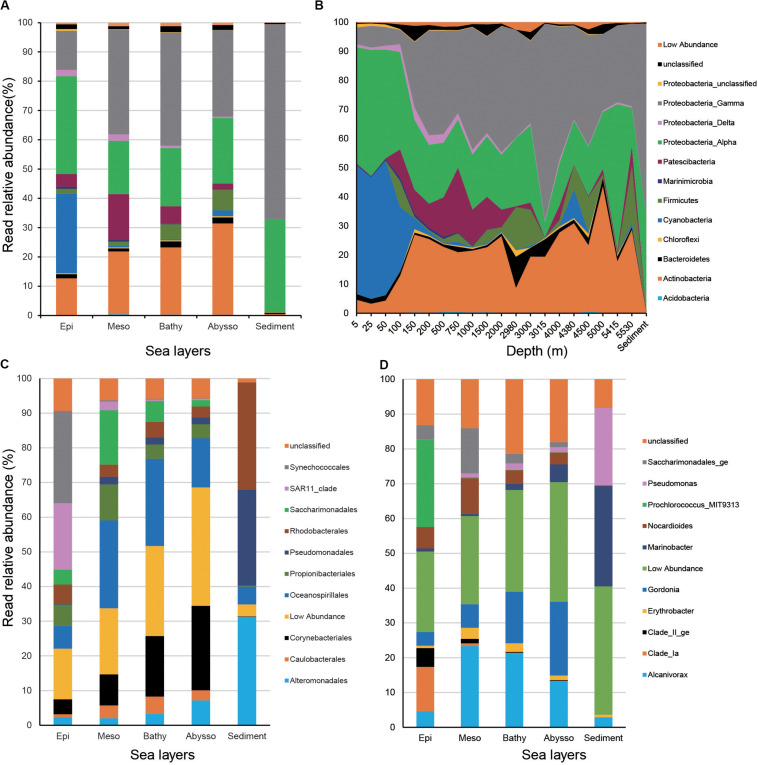
Relative read abundances of the main taxonomic groups (mainly at the levels of phylum) **(A)** and depths **(B)**, and top 10 orders **(C)** and genera in different layers of the water column **(D)**.

Relative read abundance of Proteobacteria increased from surface waters to bathypelagic zones and peaked in benthic sediments (Figures 3A,B). Relative read abundance of most Gammaproteobacteria (i.e., Alteromonadales, Oceanospirillales, Pseudomonadales, and Caulobacterales) increased significantly with increasing depth (*p* < 0.05), while that of alphaproteobacterial SAR11 clade decreased strongly (*r* = −0.85, *p* < 2.2e-16) (Figure 3C and Supplementary Table S4). Actinobacteria was significantly enriched in the deep-zone communities (*r* = 0.46, *p* < 4.62e-06). As anticipated, members of Cyanobacteria, mainly represented by the order Synechococcales, exhibited a significant decrease from the surface to the abyssopelagic zones (*r* = −0.80, *p* < 2.2e-16). The above trends were also reflected at the genus level, since relative read abundance of *Gordonia*, *Marinobacter*, *Alcanivorax*, *Pseudomonas* (Gammaproteobacteria), and *Erythrobacter* (Alphaproteobacteria) significantly increased with depth (*p* < 0.05), while, conversely, members of Clade_Ia, Clade_II_ge (SAR11), and *Prochlorococcus* exhibited opposite depth distribution (*p* < 0.001) (Figure 3D and Supplementary Table S4).

**FIGURE 4:**
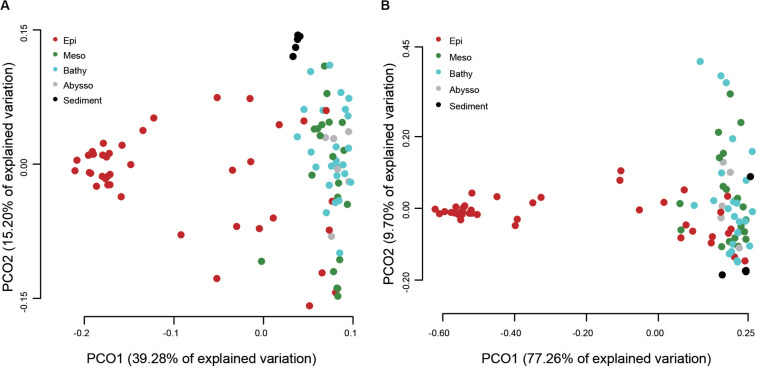
Principal coordinate analysis (PCoA) plot of different water column zones based on the weighted UniFrac distances **(A)** and the predicted functional dissimilarities **(B)**.

A PCoA analysis demonstrated that sediment samples formed a clearly distinct group (Figure 4A). Most of the samples from epipelagic waters were distinct from that of the deeper waters, but no significant differences were found between meso-, bathy-, and abyssopelagic zones (*p* > 0.05). According to the *adonis* test, the difference of bacterial community composition in the water column was mainly explained by depth (*R*^2^ = 0.33, *p* = 0.001), followed by layer of the water column (*R*^2^ = 0.31, *p* = 0.001) and station (*R*^2^ = 0.11, *p* = 0.027). A Mantel test also indicated that depth significantly influenced the bacterial community composition within the water column (*r* = 0.51, *p* = 0.001) (Table 1).

**TABLE 1:** Pearson’s correlations between environmental variables and bacterial α- and β-diversity (Mantel test).

Sea layer	Environmental variable	OTU richness	Shannon index	Community composition	Functional structure
Epipelagic zone	Depth	0.72***	0.48**	0.12	0.25**
	DO	−0.70***	−0.44**	0.67**	0.74**
	Temperature	−0.68***	−0.50***	0.63**	0.70**
	Salinity	0.73***	0.45**	0.28**	0.30**
	NO_3_^–^	0.73***	0.52***	–0.06	0.02
	PO_4_^3–^	0.22	0.31*	–0.12	–0.06
	SiO_3_^2–^	–0.14	–0.001	0.14	0.10
Below depth of 200 m	Depth	−0.35*	0.008	0.14**	–0.002
Whole water column	Depth	−0.42***	0.20	0.51**	0.66**

*p < 0.05; **p < 0.01; ***p < 0.001.

### The Functional Structure Is Shaped by Depth

**FIGURE 5:**
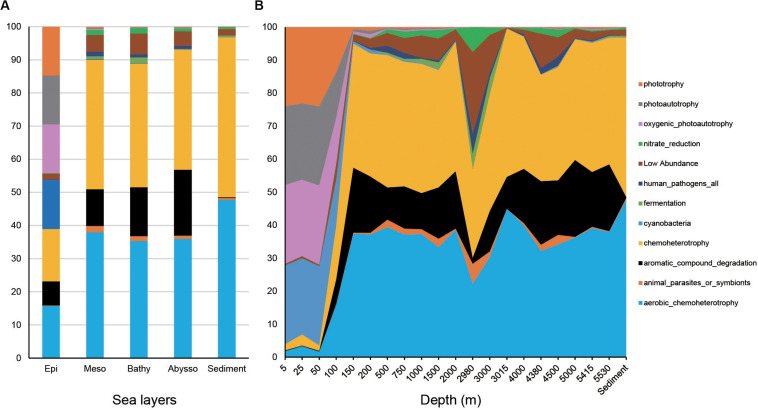
Read abundances of predicted functional groups in different water column zones **(A)** and depths **(B)**.

Among the 1,004 OTUs generated by the QIIME pipeline, 600 could be assigned to at least one functional group using the FAPROTAX database and a total of 41 functional groups were recognized (Supplementary Table S5). The functional composition of bacterial communities was more diverse in the epipelagic zone than other water zones and the sediment cores (Figure 5A). Among the top 11 groups, four groups (i.e., aerobic chemoheterotrophy, aromatic compound degradation, chemoheterotrophy, and nitrate reduction) were significantly enriched with depth (Figure 5B and Supplementary Table S4). Sediment samples were dominated by aerobic chemoheterotrophy and chemoheterotrophy, accounting for 47.7 and 48.26%, respectively. As anticipated, the four functional groups associated with light (i.e., cyanobacteria, oxygenic photoautotrophy, photoautotrophy, and phototrophy) decreased significantly with increasing depth.

The putative functional structure of bacterial communities also separated most samples of the epipelagic zone from those of the deeper zones and sediment cores (Figure 4B). Unlike the taxonomic composition, functional composition of sediment bacteria could not be clearly distinguished from other deep water layers. Depth was the parameter that better explained (*R*^2^ = 0.59, *p* = 0.001) the variation of functional structure in the water column, followed by layer of the water column (*R*^2^ = 0.46, *p* = 0.001). The variation was not significantly linked to station (*p* > 0.05). A Mantel test further supported a significant impact (*r* = 0.66, *p* = 0.001) of depth on the functional structure of the bacterial communities (Table 1).

### Environmental Variables Influenced Bacterial Diversity in the Epipelagic Zone

Vertical hydrographic profiles of the seven investigated locations were predicted using data extracted from the WOA13 dataset from the surface water to 200 m depth (i.e., the epipelagic layer) (Supplementary Table S1). Both temperature and DO decreased with increasing depth, whereas salinities for all locations reached the highest values (34.62–35.09‰) at about 150 m depth. Usually, concentrations of NO_3_^–^ and PO_4_^3–^ increased with increasing depth and a larger increase in SiO_3_^2–^ concentration occurred at a depth of 50 m. The two locations, QB10 and QB11, which were located near the Philippines, showed relatively higher concentrations of NO_3_^–^ (5.31, 5.08 μmol/L) and PO_4_^3–^ (0.88, 0.88 μmol/L) in the surface waters compared to other locations.

In the epipelagic zone, both bacterial richness and Shannon indices significantly increased with depth. Bacterial α-diversity was negatively correlated with temperature and DO (Table 1) but increased with salinity and NO_3_^–^ concentration. Bacterial community composition was not significantly influenced by depth, but by DO, temperature, and salinity. Partial Mantel tests revealed a combined effect of temperature and DO on bacterial community structure. After removing the influence of temperature or DO, a sharp decrease of Mantel’s *r* values of DO or temperature occurred (Supplementary Table S6). This combined effect was also observed using functional structure data. Moreover, a dependent influence of depth related to DO was detected (*r* = 0.118, *p* = 0.06). Salinity likely had significant and independent effects on both community and functional structure of the epipelagic zone (Supplementary Table S6). However, we should acknowledge the limits of these above correlations estimated in the epipelagic layer, as the environmental variables used refer to historical data, not in-situ data, from CTD.

## Discussion

Consensus on the generality of trends in prokaryotic richness along vertical gradients has not yet been reached. Our results showed a trend of OTU richness from the surface to the abyssopelagic zone, with maximal OTU richness occurring at depths ranging from 100 to 200 m. Below the epipelagic zone, with the increase of depth, light penetration declined gradually, accompanied with a decrease in organic matter (Lønborg et al., 2016). From the mesopelagic zone to deeper layers, bacterial diversity decreased with depth, possibly because microbial niches are restricted by the availability of light and labile organic matter (Bryant et al., 2016). The trend observed in this study is in part consistent with the findings observed in the Mediterranean Sea (Pommier et al., 2010) and Arctic Ocean (Wilson et al., 2017), as well as across the global ocean (Sunagawa et al., 2015), where species richness increases from the surface to the mesopelagic zone. In the South China Sea, the maximal bacterial diversity was observed at 1,000 m depth (Tseng et al., 2015). Recently, a study showed that bacterial diversity on particles increased with depths up to 2,300 m in the NW Mediterranean Sea (Mestre et al., 2017). These observed discrepancies may be due to either the different hydrographic parameters of the locations investigated or the technical distinctions in the sample processing.

As expected, our taxonomic profiles revealed that members of Cyanobacteria (mainly represented by *Prochlorococcus*) and SAR11 clade dominated the surface of the water column, which is similar to previous studies conducted in the western Pacific (Liang et al., 2016), the North Pacific gyre areas (Delong et al., 2006), and Arctic water (Hamdan et al., 2013). Meanwhile, members of *Prochlorococcus* and SAR11 clade are suggestive of depth-variable adaptation to light intensity and nutrient availability (Rocap et al., 2003; Delong et al., 2006). Our data might provide further evidence on the strongly negative correlations between relative abundance of the two groups and depth. *Prochlorococcus* microbes are considered as the major primary producers in the ocean, responsible for a large percentage of the photosynthetic production of oxygen (Kaiser and Benner, 2012). Here, we should note that the reverse primer used in this study is the original 806R primer (Muyzer et al., 1993; Caporaso et al., 2012) and could cause lower detection of SAR11 clade due to its mismatch with this clade compared with the new 806R primer modified by Apprill et al. (2015). On the other hand, Actinobacteria, Gammaproteobacteria, and Firmicutes increased their relative representation in the deep-sea prokaryotic community compared to surface waters of the western Pacific. This is consistent with previous studies conducted in deep-water masses of the North Atlantic (Sogin et al., 2006; Wu et al., 2013), the Greenland Sea (Zaballos et al., 2006), and global seawater ecosystems (Zinger et al., 2011), where deep-water samples showed higher relative sequence abundance of these taxonomic groups than surface-water. The adaptive advantage of these microbes may let them thrive under extreme oligotrophic conditions of the deep ocean (Gärtner et al., 2011; Wu et al., 2013). Alternatively, this can be explained partially by their obligate growth requirement for elevated hydrostatic pressures (Yayanos, 1995).

Patescibacteria are part of the Candidate Phyla Radiation with ultra-small genomes and have a presumed symbiotic or parasitic lifestyle (Sánchez-Osuna et al., 2017; Lemos et al., 2019). Members of Patecibacteria were found to be enriched in the disease lesions of coral species from Thailand (Roder et al., 2014) and the Florida Reef Tract (Meyer et al., 2019). In the sediments of the Challenger Deep in the Mariana Trench, the Patecibacteria phylum was one of the most dominated bacterial phyla (>1% relative read abundance) (Cui et al., 2019). Here, we observed a high relative abundance of Patescibacteria in the water column of the Western Pacific Ocean, especially at depths ranging from 200 to 1,500 m. These observations raise questions on their adaptation to the marine environments they inhabit and the ecological functions they probably perform.

As the most dominant phylum in deep-sea sediments, Proteobacteria has already been described in many previous studies (e.g., Durbin and Teske, 2011; Bienhold et al., 2016; Walsh et al., 2016). Our results showed that Gamaproteobacteria was most dominant in sequence abundance, followed by Alphaproteobacteria in sediments from the Philippine Basin. The predominance of the Gamaproteobacteria over the Alphaproteobacteria and Deltaproteobacteria was detected in deep-sea sediments at a global scale (Bienhold et al., 2016), as well as the regional study conducted in the South Atlantic Ocean (Schauer et al., 2010). Alphaproteobacteria was found to be more abundant than Gemmatimonadetes in sedimentary communities of the South Pacific (Durbin and Teske, 2011), Equatorial Pacific, and North Pacific (Walsh et al., 2016). We also found that Actinobacteria occurred with small proportions in sedimentary communities, which contrasts with the global study that documented Actinobacteria as the second most abundant group in marine sediments (Bienhold et al., 2016). This study also clearly documents a difference in community composition between sediments and seawater column, which is consistent with both global (Bienhold et al., 2016) and regional studies, e.g., Equatorial Pacific and North Pacific (Walsh et al., 2016), South Pacific (Durbin and Teske, 2011). Here, we admit that the comparison of bacterial communities between sediments and water columns were not sampled from the water column overlaying those specific sediments but in different areas.

Stratified microbial communities show significant differences in gene content and metabolic pathways (Delong et al., 2006). In the epipelagic zone, the groups closely associated with light and oxygen, such as phototrophy, photoautotrophy, and oxygenic photoautotrophy, are abundant, which is generally consistent with previous surveys in the water column (Delong et al., 2006). On the contrary, some groups (e.g., *Gordonia*, *Marinobacter*, *Alcanivorax*, *Pseudomonas*, and *Erythrobacter*), capable of aerobic chemoheterotrophy and chemoheterotrophy, are overrepresented in deeper zones and sediment where both light and oxygen are often limiting (Lønborg et al., 2016). This differs from the previous study that reported functional groups of fermentation or nitrate respiration dominated in the mesopelagic zone across global oceans based on metagenomic sequencing (Louca et al., 2016). However, we should note that this difference may be due to PCR bias in amplicon sequencing. The quantity and quality of the organic carbon decreases with depth in the ocean (Jiao et al., 2010; Hansell, 2013; Giering et al., 2014). Heterotrophic microbial activity is likely sustained by organic carbon supplied by primary production in the euphotic zone. As a result, the rates of microbial growth, production, and microbial-mediated carbon cycling will decline with depth (De Corte et al., 2012; Li et al., 2014). The increasing relative abundance of heterotrophic groups along depth may effectively compensate for the microbial activity due to the lack of organic carbon in deep waters, confirming a prevalent heterotrophic lifestyle for deep-sea microbes (Martín-Cuadrado et al., 2007).

Using taxonomic and functional profiling of prokaryotic communities from the *Tara* Oceans expedition based on the shotgun 16S rRNA gene, Louca et al. (2016) found that environmental variables were generally less correlated to relative taxon abundances at the community level than to relative abundance of functional groups. In the South China Sea, the active bacterial community displayed higher environmental sensitivity than the total bacterial community (Zhang et al., 2014). Similarly, our data showed that several environmental factors, including DO, depth, temperature, and salinity, were found to have a closer relationship with functional structure than that of taxonomic community in the epipelagic zone. The high sensitivity of functional structure to environmental factors might be advantageous for adapting to the dynamic physic-chemical gradient in the epipelagic water of the open sea.

Spatially limited dispersal is another key factor shaping microbial taxonomy and functional composition (e.g., Zhang et al., 2014; Sunagawa et al., 2015). In this study, however, station showed a significant effect on bacterial taxonomic composition, but not on the functional composition. This may be explained by the fact that distantly related microbes can often perform similar metabolic functions (Martiny et al., 2015). On the other hand, no significant difference among the deeper zones (i.e., meso-, bathy-, and abyssopelagic zones) in both bacterial taxonomic and functional compositions was detected in our dataset. In the ocean, sinking particles, known as the particulate organic matter flux from the euphotic zone (Delong et al., 2006; Kaiser and Benner, 2012), is assumed as an important source of carbon impacting microbial communities (Pelve et al., 2017). In addition, these particles are found to be hotspots for microbial life in the deep ocean (Bochdansky et al., 2017; Pelve et al., 2017), which would greatly shape the community structure of deeper waters (Frank et al., 2016; Lønborg et al., 2016). Thus, sinking particles occupied by various microbial communities may in part contribute to homogenous communities of bacteria among the deeper zones in the present study.

## Conclusion

This study revealed the vertical distribution patterns in taxonomic compositions of bacterial assemblages from the surface to abyssopelagic zones and even sediments of the western Pacific. Additionally, predictions of ecological functions from taxonomic profiles among different zones and benthic sediments underline hints into the ecology of marine prokaryotes and their adaptation with increasing depths. Compared with taxonomic composition, predicated functional structure exhibited closer relationships with environmental variables in the epipelagic zone, highlighting a high sensitivity to the dynamics in a physic-chemical gradient. However, the associations presented here do not necessarily suggest a causal effect of environmental variables on functional structure, which will require further investigation using metatranscriptomics or other functional analyses.

## Data Availability Statement

The SRA accession number for sequencing data can be found in the NCBI under accession number SRP250935.

## Author Contributions

MW processed part of the raw data, programmed part of the bioinformatic analysis, drafted part of the manuscript, approved the final version of the manuscript, submitted the raw data to SRA, and submitted the manuscript to Frontier in Microbiology. YM performed partial experiment of the work. CF performed partial sampling of the work. LC drafted most part of the manuscript, and revised the manuscript. WL conceived and designed the work that led to the submission, programmed most of the bioinformatic analysis, analyzed data, drafted part of the manuscript and approved the final version of the manuscript. All authors contributed to the article and approved the submitted version.

## Conflict of Interest

The authors declare that the research was conducted in the absence of any commercial or financial relationships that could be construed as a potential conflict of interest.

## Supplementary Material

FIGURE S1 Rarefaction curves for each sample using the data after resampling to the lowest number of bacterial reads (25,345) in any individual sample.

TABLE S1 Information of samples on water depth, salinity, temperature, dissolved oxygen and nutrients, and alpha diversity.

TABLE S2 Primer identifier barcodes of 97 samples.

TABLE S3 Annotation of OTUs and distribution in different samples.

TABLE S4 Pearson’s correlations between bacterial taxonomic and functional diversity and environmental variables.

TABLE S5 Functional groups classified using FAPROTAX database.

TABLE S6 Mantel/partial Mantel tests of taxonomic and functional compositions.
