# Supplementary figures and images for: Diversity of Pelagic and Benthic Bacterial Assemblages in the Western Pacific Ocean
Source: Front Microbiol. 2020 Sep 10;11:1730. doi: 10.3389/fmicb.2020.01730 (PMC7533643; doi:10.3389/fmicb.2020.01730)

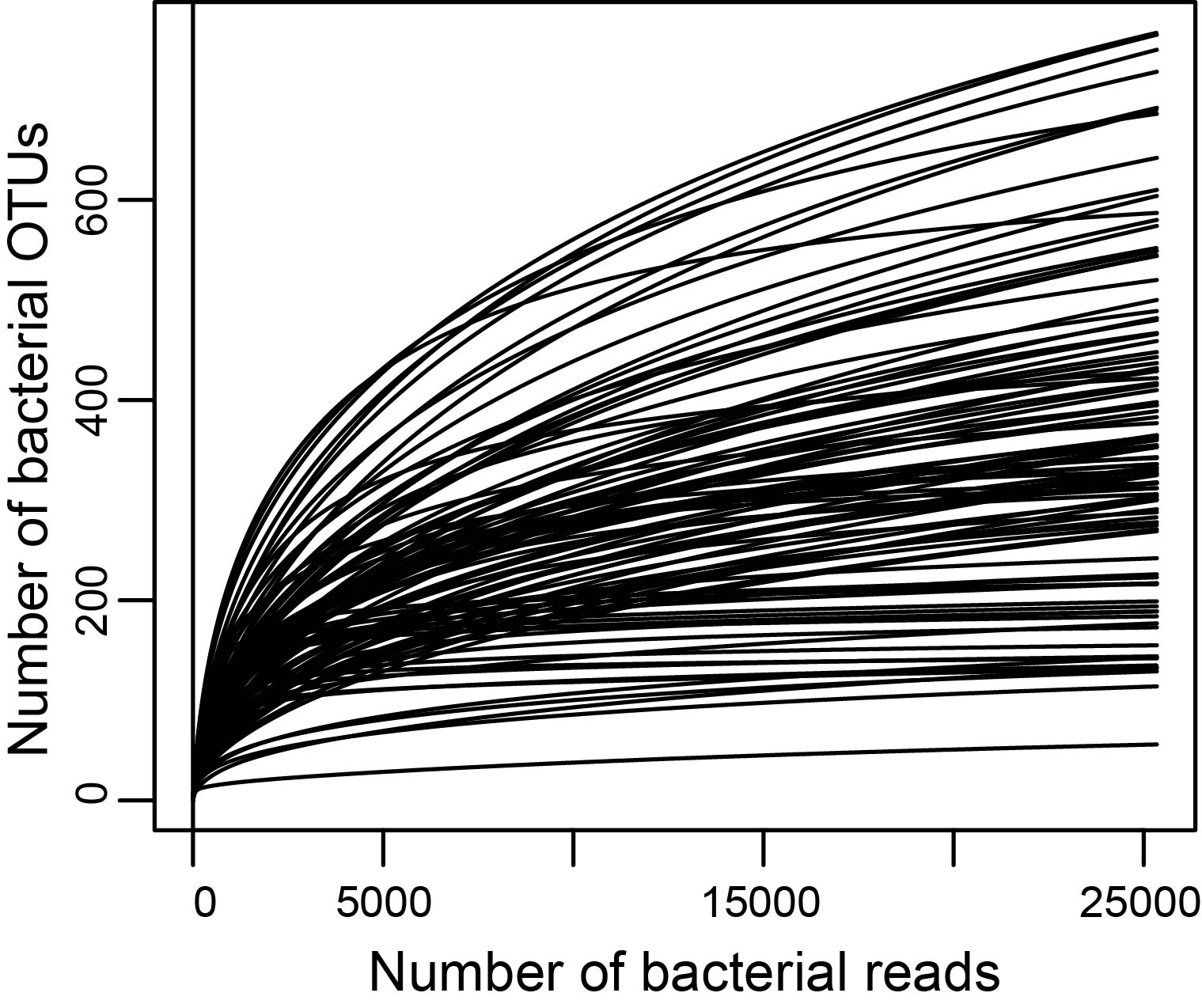

Supplement: FIGURE S1 — Rarefaction curves for each sample using the data after resampling to the lowest number of bacterial reads (25,345) in any individual sample. [file Image_1.JPEG]
